# Authentic and Ectopically Expressed MRGPRX2 Elicit Similar Mechanisms to Stimulate Degranulation of Mast Cells

**DOI:** 10.3390/cells10020376

**Published:** 2021-02-12

**Authors:** Pia Lazki-Hagenbach, Hydar Ali, Ronit Sagi-Eisenberg

**Affiliations:** 1 Department of Cell and Developmental Biology, Sackler Faculty of Medicine, Tel Aviv University, Tel Aviv 69978, Israel; piahagenbach@gmail.com; 2 Department of Basic and Translational Sciences, School of Dental Medicine, University of Pennsylvania, Philadelphia, PA 19104, USA; alih@upenn.edu

**Keywords:** mast cells, Mas-related G-protein-coupled receptors, MRGPRX2, degranulation, RBL-2H3

## Abstract

The identification of the Mas-related G-protein-coupled receptors (Mrgpr) as targets of diverse stimuli of mast cells (MCs), including neuropeptides and pseudo-allergy causing drugs, has placed these receptors at a prime position in MC research. However, the species-dependent diversity of these receptors raises the need for an adequate model for investigating the human MRGPRX2 receptor. RBL-2H3 cells, stably transfected with MRGPRX2 (RBL-MRGPRX2), are increasingly used for this purpose. Therefore, we investigated whether ectopically expressed MRGPRX2, in rat MCs, recapitulates its authentic signaling. To this purpose, we performed a broad comparative study of the responses of human LAD-2 MCs that express MRGPRX2 endogenously, and RBL-MRGPRX2 cells to compound 48/80, substance P and vancomycin, three proto-type ligands of MRGPRX2. We demonstrate that both models share similar dose–response relationships, kinetics and sensitivities to a wide range of signaling targeting drugs. Therefore, our results indicate that ectopically expressed MRGPRX2 preserves the signaling pathways employed to evoke human MC degranulation, which we show to rely on ERK1/2 MAP kinases, phospholipase C (PLC) and autophagy-related signaling. Importantly, we also show that the underlying mechanisms of MRGPRX2-triggered MC degranulation in either LAD-2 or RBL-MRGPRX2 cells are different from those elicited by its rodent orthologs.

## 1. Introduction

Alongside their role in immunoglobulin E (IgE)-triggered allergy [1], mast cells (MCs) have long been recognized as sentinel cells, due to their strategic location in the skin and at mucosal surfaces and their IgE-independent responses to multiple stressors [2,3]. In particular, intriguing in this regard are the responses of a subset of MCs to a large range of positively charged molecules, including neuropeptides, hormones and antimicrobial peptides [4], as well as polyamines, toxins and multiple FDA-approved drugs [5,6,7,8,9,10]. Exposure to this class of stimuli results in the rapid release of preformed inflammatory mediators, such as histamine, proteases and glycosaminoglycans, that are stored in the MC secretory granules (SGs) [11,12,13], followed by the release of metabolites of arachidonic acid and some cytokines [14,15]. Jointly, these mediators cause neurogenic inflammation [16,17,18], as well as allergic and pseudo-allergic reactions.

The mechanism of action of this class of MC stimuli has remained enigmatic until the recent identification of the Mas-related G-protein-coupled receptors (Mrgprs) as their cellular targets [7,9,19,20]. However, while this recognition has solved the riddle of this MC pathway, it has raised a new challenge. Mrgprs comprise a multi-gene family of GPCRs that have expanded, through evolution, in a species-dependent manner [21,22]. Thus, the A-H subfamilies exist only in rodents, while subfamily X exists only in humans and other primates [20]. Therefore, rodent MCs might not constitute an adequate model for predicting responses of human MCs to this class of stimuli. Given the difficulty in isolating and cultivating MCs from human tissue biopsies, three model systems are often used to overcome this difficulty. The latter include in vitro differentiated MCs that are derived from human peripheral or cord blood, the LAD-2 human mast cell line, and RBL-2H3 cells, a rat mast cell line, that are stably transfected with human MRGPRX2 [21]. Out of these models, RBL-MRGPRX2 cells have the advantage of higher transfection efficiency and growth as adherent cultures in multi-well plates, making them an ideal model for functional genomics analyses and high-throughput screening. Moreover, the fact that naïve RBL-2H3 cells are Mrgpr-deficient and, accordingly, lack any response to Mrgpr-activating ligands allows us to distinguish responses that are exclusively mediated by MRGPRX2, from responses that might be mediated by the canonical receptors that some of these ligands have [6,7,23]. However, along with these advantages, the main disadvantage of the RBL-MRGPRX2 cells is that, unlike the human derived MCs that express MRGPRX2 endogenously, this receptor is ectopically expressed in rat MCs, in the RBL-MRGRX2 model. The question, therefore, arises whether the human receptor indeed recapitulates the underlying mechanisms utilized by the endogenous receptor to trigger human MC degranulation, or whether it acquires the characteristics of the rodent receptor, by interacting with the downstream effectors of its rodent orthologs. Since RBL-MRGPRX2 cells are increasingly used as a model, we set out to address this question by undertaking a comparative study of MRGPRX2 responses in LAD-2 and RBL-MRGPRX2 MCs. We also compared these responses to the responses of mouse peritoneal MCs that endogenously express the murine ortholog. Here we show that MRGPRX2 retains its authentic mechanisms for inducing secretion from MCs also in RBL-MRGPRX2 cells. Our results shed light on these mechanisms and demonstrate that they are different from those elicited by its rodent orthologs.

## 2. Materials and Methods

### 2.1. Antibodies and Reagents

Anti-human MRGPRX2 antibody (cat # 359002, 1:100 dilution) was from Biolegend (San Diego, CA, USA), anti-HA.11 Epitope Tag antibody was from Biolegend (cat # 901513), secondary 647 (cat # 61057-H647, 1:200 dilution) was from Anaspec (Fremont, CA, USA), anti phospho extracellular signal-regulated kinase (ERK)1/2 (cat # M8159, 1:10,000 dilution) was from Sigma-Aldrich and anti-total ERK2 (cat # sc-154, 1:1000 dilution) was from Santa Cruz Biotechnology (Dallas, TX, CA, USA). Horseradish peroxidase-conjugated goat anti-mouse (cat # 115-035-166) and anti-rabbit (cat # 115-035-003) IgG were from Jackson ImmunoResearch Laboratories (West Grove, PA, USA). Compound 48/80 (cat # C2313), substance P (cat # S6883), *p*-nitrophenyl-*N*-acetyl-β-d-glucosaminide (cat # N9376) and o-phthalaldehyde (cat # P0657) were purchased from Sigma-Aldrich (St Louis, MO, USA). Vancomycin Hydrochloride (cat # 195540) was purchased from MP Biomedicals, LLC (Solon, OH, USA). Drugs used in this study are listed in Table 1.

### 2.2. Cell Culture

RBL-MRGPRX2 cells were generated as previously described [24]. RBL-MRGPRX2 cells and their parental RBL-2H3 (herein referred to as RBL) counterparts were maintained at 37 °C, in a humidified incubator with 5% CO_2_, in adherent cell cultures in low-glucose DMEM (cat # 01-050-1A, Biological Industries, Beit-Haemek, Israel) supplemented with 10% FBS (cat # 12657, GIBCO, Grand Island, NY, USA), 2 mM L-Glutamine (cat # 03-020-1A, Biological Industries), 100 μg/mL streptomycin and 100 U/mL penicillin and 12.5 U/mL nystatin (Biological Industries, Beit-Haemek, Israel), except for RBL-MRGPRX2 cells that were additionally supplemented with 1 mg/mL of G418 (cat # A1720, Sigma Aldrich, St Louis, MO, USA). LAD-2 cells (a kind gift from Dr. A.S. Kirshenbaum and Dr. D. Metcalfe, Laboratory of Allergic Diseases, National Institute of Allergy and Infectious Diseases, National Institutes of Health, Bethesda, MD, USA) were cultured in StemPro-34 (cat # 10640-019, GIBCO) supplemented with 1× StemPro-34 Nutrient, 2 mM L-Glutamine (cat # 03-020-1A, Biological Industries), 100 U/mL penicillin and 100 μg/mL streptomycin (cat # 03-032-1B, Biological Industries), and 100 ng/mL hSCF (cat # 300-07, Peprotech, Rocky Hill, NJ, USA).

### 2.3. Cell Transfection

Transient transfection was performed as previously described [24,25]. Briefly, RBL cells (1.5 × 10^7^) or LAD-2 cells (5 × 10^5^) were transfected with 30 μg of Neuropeptide Y fused to monomeric red fluorescent protein (NPY-mRFP) cDNA by electroporation at 300 V for 20 ms, using an ECM 830 electroporator (BTX, Holliston, Mass, USA). The cells were immediately replated in 24-well (1 × 10^5^ cells/well) tissue culture dishes containing growth medium and analyzed after 24 h.

### 2.4. Isolation of Mouse Peritoneal MCs

Mouse peritoneal cells were isolated by intraperitoneal lavage from female C57BL mice weighing ~20 g. The peritoneal cavity was then opened by a midline incision. Then, 10 mL sterile PBS was injected into the peritoneal cavity of each mouse, and the abdomen was massaged for 30 s, to cause circulation of the PBS in the peritoneum, and the fluid was collected. The fluid from several mice was combined, spun down at 300 g for 5 min and resuspended at 3.5 × 10^6^ to 5 × 10^6^ cells/mL. The peritoneal mixture contained ~2.5 × 10^6^ cells/mouse, out of which 4–5% of cells were identified as MCs by toluidine blue staining. All experiments were approved by the Institutional Animal Care and Use Committee at Tel Aviv University (# 01-20-086).

### 2.5. Mast Cell Activation

RBL or RBL-MRGPRX2 cells were either seeded onto 96-well plates at 0.4 × 10^5^ cells/well for secretion assays or onto 6-well plates at 5 × 10^5^ cells/well for Western blot analyses. The following day, cells were washed 3 times in Tyrode’s buffer (10 mmol/L HEPES [pH 7.4], 130 mmol/L NaCl, 5 mmol/L KCl, 1.8 mmol/L CaCl_2_, 1 mmol/L MgCl_2_, 5.6 mmol/L glucose and 0.1% BSA) and treated with the indicated concentrations of stimuli in the absence or presence of inhibitors, in Tyrode’s buffer, at 37 °C, in a final volume of 100 µL for secretion assays and 1 mL for Western blot analyses. 

LAD-2 cells were resuspended in Tyrode’s buffer, at a concentration of 5 × 10^5^ cells/mL for secretion assays and at 1 × 10^6^ cells/mL for Western blot analyses, and triggered at final volumes of 200 µL and 1 mL, respectively. 

Mouse peritoneal cells were resuspended in Tyrode’s buffer, at 3.5 × 10^6^ to 5 × 10^6^ cells/mL, and triggered for secretion in a final volume of 200 µL. 

### 2.6. β-Hexosaminidase Release Assay

β-hexosaminidase activity was determined as previously described [26]. Briefly, 20 μL aliquots of supernatants and cell lysates derived from cells triggered as indicated were incubated for 90 min, at 37 °C, with 50 μL substrate solution consisting of 1.3 mg/mL p-nitrophenyl-*N*-acetyl-β-*D*-glucosaminide in 0.1 M citrate (pH 4.5). Subsequently, reactions were stopped by the addition of 180 μL 0.2 M glycine (pH 10.7), and optical density was measured at 405 nm, by an absorbance microplate reader (Infinite F50, Tecan, Männedorf, Switzerland).

### 2.7. Histamine Release Assay

The amount of histamine released was determined as previously described, using the o-phthalaldehyde (OPT) fluorometric method [27]. Cells were incubated for 30 min with vehicle or indicated inhibitor, followed by their stimulation by either c48/80 (10 μg/mL) or SP (100 μM) for a further 30 min. Then, 100 µL of supernatants and cell lysates derived from these cells were incubated with 20 µL of 1 N NaOH and 5µL of OPT (10 mg/mL), for 4 min. The reaction was terminated by the addition of 10 µL of 3N HCl. Fluorescence was measured by using a SynergyHTX_2018 microplate reader (λ excitation 340 nm, λ emission 440 nm).

### 2.8. Flow Cytometry Analysis

RBL-MRGPRX2 cells were directly stained with anti-human MRGPRX2 Ab (20 min, room temperature, 1:100 dilution), washed and subsequently stained with secondary 647 conjugated goat anti-mouse IgG (H + L), highly cross-adsorbed, for 20 min, at room temperature, in the dark (1:200 dilution). Cells were analyzed by flow cytometry, using a CytoFLEX LX flow cytometer (Beckman Coulter, Indianapolis, IN). Data were analyzed by using the FlowJo™ Software Version 10 (Treestar, Ashland, OR, USA). 

### 2.9. Immunostaining and Laser Confocal Microscopy Analysis

RBL-MRGPRX2 and LAD-2 cells grown on untreated (RBL-MRGPRX2) or fibronectin (cat # F1141, Sigma Aldrich, St Louis, MO, USA) coated (LAD-2) 12 mm round glass coverslips (thick #1; Thermo Scientific, Menzel-Gläser, Saarbrücken, Germany) were washed 3 times with ice-cold PBS and fixed for 20 min, at room temperature, with 4% paraformaldehyde (catalogue # 15,710; Electron Microscopy Sciences, Hatfield, PA, USA) in PBS. Fixed cells (RBL-MRGPRX2 or LAD-2) were incubated for 1 h, at room temperature, with the desired primary antibodies, followed by three washes and a 1 h incubation, with the appropriate secondary antibody. After washing, cells were mounted (cat # E18-18; Golden Bridge Life Science, Mukilteo City, WA, USA) and analyzed with a Leica SP5 laser scanning confocal microscope (Leica, Wetzlar, Germany) equipped with a Hybrid Detector (HyD), using a ×63 oil/1.4 NA objective for imaging. Co-localization analysis of NPY-mRFP with immunostained MRGPRX2 (M1) was quantified as the Manders’s coefficient with Costes’s automatic threshold, using the JaCoP plugin of the extended ImageJ version Fiji [28,29,30].

### 2.10. Western Blot Analyses

RBL-MRGPRX2 or LAD-2 cells were lysed by the addition of a lysis buffer (0.15 M sucrose, 80 mM β-glycerophosphate, 1% Triton X-100, 2 mM EDTA, 2 mM EGTA, 2 mM Na_3_VO_4_, 10 mM NaPPi, 1 mM PMSF and 1:25 dilution of protease inhibitor cocktail (Roche)) and incubated for 10 min, on ice. The lysates were then centrifuged for 15 min, at 14,000 × g. Whole cell lysates, normalized according to cell number, were mixed with Lämmli sample buffer (1:5), boiled for 10 min and subjected to SDS-PAGE, using 10% polyacrylamide gels, and transferred electrophoretically to nitrocellulose membranes. Blots were blocked for at least 30 min in TBST (10 mM Tris-HCl, pH 8.0, 150 mM NaCl and 0.05% Tween-20) containing 5% skim milk, followed by overnight incubation at 4 °C, with the desired primary antibodies. Blots were subsequently washed three times with TBST and incubated for 1 h, at room temperature, with the secondary HRP-conjugated antibodies. Immunoreactive bands were visualized by enhanced chemiluminescence according to standard procedures. The intensity of immunoreactive bands was quantified using the ImageJ software, and the relative pixel densities (phosphorylated/total) were calculated.

### 2.11. RNA Purification and Quantitative Real-Time PCR

Total cellular RNA was extracted with the GENEzol TriRNA Pure Kit (cat # GZXD200; Geneaid, New Taipei, Taiwan), according to the manufacturers’ protocol. The cDNA was generated by using 2 μg of total RNA and high-capacity reverse transcriptase (Applied Biosystems, Foster City, CA, USA) in a total volume of 20 μL. The cDNA was assessed by using real-time PCR (StepOne; Applied Biosystems, Foster City, CA, USA) with Power SYBR Green PCR Master Mix (cat # 4367659; Applied Biosystems, Foster City, CA, USA) and analyzed by using StepOne V.2.3 software. As a reference gene, rat or human GAPDH (rGAPDH and hGAPDH, respectively) was amplified. Cycle threshold was determined for hMRGPRX2, rMRGPRB3 and for their respective reference gene (hGAPDH or rGAPDH). Relative expression was calculated by the 2ΔΔCT method [31]. Primer sequences are listed in Table 2.

### 2.12. Statistical Analysis 

Data were analyzed by using GraphPad Prism Version 8.3.0 for Windows, (GraphPad Software, La Jolla, CA, USA). One-way analysis of variance (ANOVA) with repeated measures, followed by Dunnett’s post-test or Student’s *t*-test, was used for comparing means, respectively. Results were considered significant with *p*-values smaller than 0.05.

## 3. Results

### 3.1. Dose and Time Dependence of Endogenous and Ectopic MRGPRX2 Responses 

**Figure 1:**
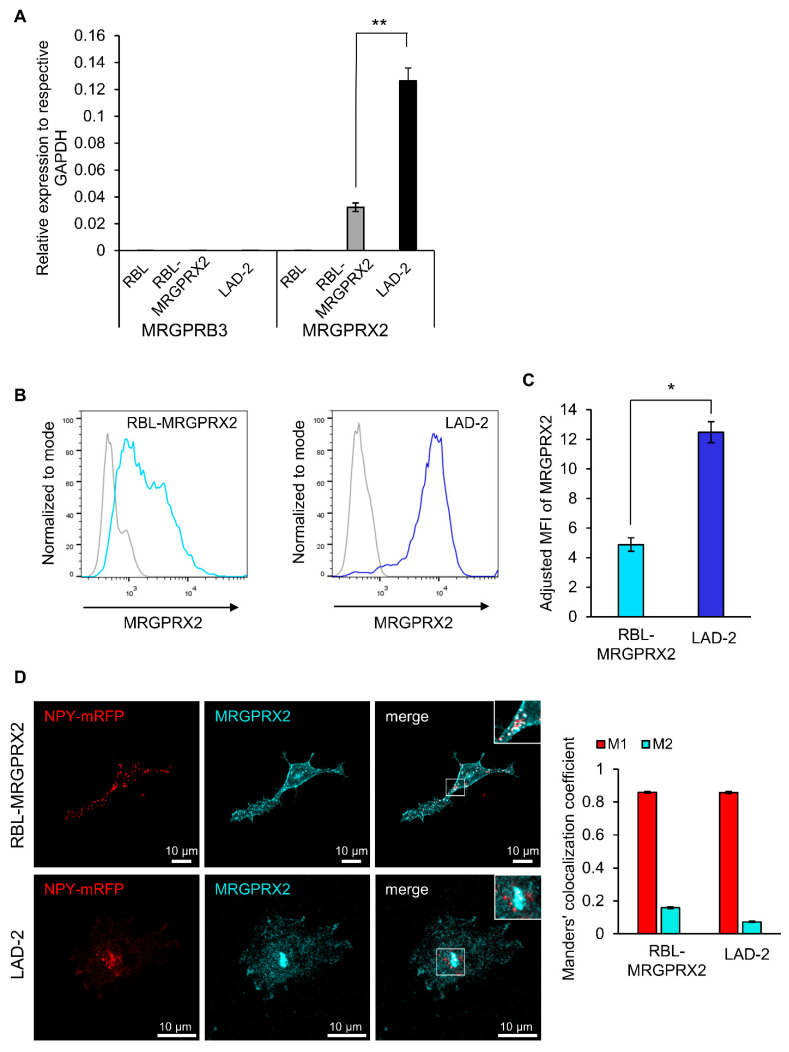
RBL-MRGPRX2 and LAD-2 cells express MRGPRX2 protein and mRNA. (**A**) Expression of human MRGPRX2 and rat MRGPRB3, relative to human and rat GAPDH, respectively, in RBL-MRGPRX2 and LAD-2 cells, was determined by quantitative qPCR. Data are the means ± SEM (n = 4). Statistical significance was determined by Student’s t-test (** *p* = 0.00619893). (**B**) RBL-MRGPRX2 and LAD-2 cells were directly stained with anti-human MRGPRX2 and analyzed by flow cytometry, for their cell surface expression of MRGPRX2. Gates in histograms (gray, control; blue, RBL-MRGPRX2/LAD-2) were set according to fluorescence minus one (FMO)-matched control staining. (**C**) The adjusted mean fluorescent intensity (MFI) of MRGPRX2 (MFI of sample/MFI of control). Data are the means ± SEM (n = 3); * *p* = 0.01290177. (**D**) RBL-MRGPRX2 and LAD-2 cells transiently transfected with NPY-mRFP cDNA were fixed and immunostained using a monoclonal antibody against MRGPRX2 (Biolegend, 1:100), followed by secondary AlexaFluor 647 conjugated goat anti-mouse IgG (H + L) (abcam, 1:500). Cells were analyzed by confocal microscopy. Bars = 10 μm. Insets are enlargements of the boxed areas. Co-localization of NPY-mRFP with immunostained MRGPRX2 was quantified for 69 LAD-2 and 91 RBL-MRGPRX2 cells from three independent experiments, using the JACoP plugin of the extended ImageJ version Fiji. Error bars represent SEM. The Manders’ co-localization coefficient analysis was employed to measure the extent of overlap of NPY-mRFP with MRGPRX2 in both cell types (Manders’ M1 NPY-mRFP = 0.8586 and 0.8571, and Manders’ M2 MRGPRX2 = 0.1586 and 0.0724, in RBL-MRGPRX2 and LAD-2 cells, respectively).

Quantitative real-time PCR analyses confirmed that RBL cells that were stably transfected with a plasmid encoding HA-MRGPRX2, i.e., RBL-MRGPRX2 cells [21], but not naïve untransfected RBL cells, express the human MRGPRX2; however, the expression level of the receptor was lower, compared to LAD-2 cells, which contained four-fold more mRNA (Figure 1A). This analysis also demonstrated that naïve RBL cells barely express (≥35—undetermined) MrgprB3, the rat ortholog of MRGPRX2 (Figure 1A). In this context, it is noteworthy that some RBL-2H3 cell lines do endogenously express MrgprB3 mRNA, and though the amount is significantly smaller than MrgprB3 expression in rat peritoneal MCs [35], some RBL cells do respond to Mrgpr-activating ligands [35,37,38]. These observations reinforce the need to examine the responsiveness of the parental RBL cells to Mrgpr ligands, prior to their transfection with MRGPRX2, to ascertain that their responses are indeed exclusively mediated by the human MRGPRX2 receptor. 

The flow-cytometry analysis confirmed that RBL-MRGPRX2 cells also expressed the receptor on their cell surface (Figure 1B), though the amount of cell surface receptor was 2.5-fold lower than surface expression in LAD-2 cells (Figure 1B,C). Visualization of immunostained cells by confocal microscopy confirmed the membrane expression of MRGPRX2, but also depicted the existence of an internal pool of MRGPRX2 in both LAD-2 and RBL-MRGPRX2 cells that was mostly confined to vesicular structures, reminiscent of the SGs (Figure 1D). To test this possibility, we performed a Manders’ co-localization analysis, to quantify the degree of co-localization between MRGPRX2 immunostaining and the fluorescence of Neuropeptide Y-fused to mRFP (NPY-mRFP). In previous studies, we showed that transfected NPY-mRFP localizes to MC SGs and is released in a regulated fashion, alongside the endogenous mediators [39]. Therefore, NPY-mRFP serves as a reliable reporter of MC SGs. Results confirmed a significant extent of overlap of NPY-mRFP with MRGPRX2 in both cell types, indicating that a fraction of the internal receptor resides in SGs (Figure 1D). In MCs, an intimate connection exists between the SGs and endosomes, whereby at least part of the SG membrane proteins, such as CD63, pass through the plasma membrane and endosomes, on their route to the SGs [40,41]. Therefore, MRGPRX2 may reach the SGs via its internalization from the plasma membrane and endosome fusion with the SG. Whether the SG-localized MRGPRX2 acquires a distinct role is presently unknown.

**Figure 2:**
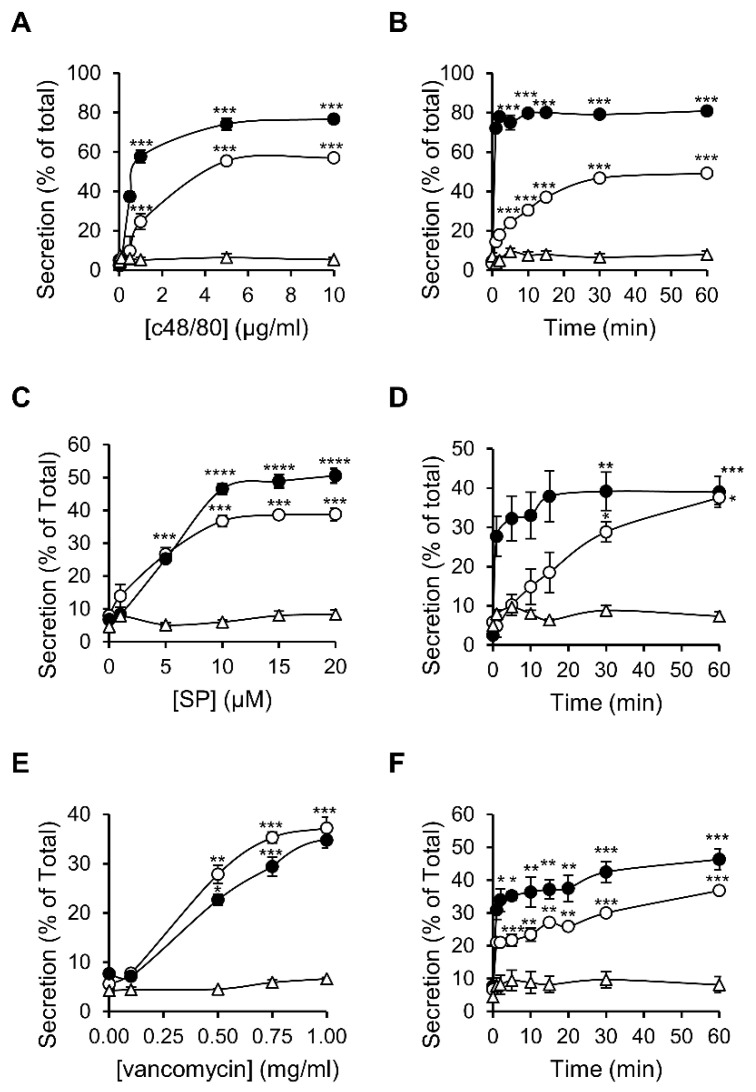
MRGPRX2-stimulated secretion shares similar dose response relationships and kinetics in LAD-2 and RBL-MRGPRX2 cells. RBL-MRGPRX2 (○), LAD-2 cells (●) or naïve untransfected RBL cells (∆) were incubated for 30 min, at 37 °C, with the indicated concentrations of c48/80 (**A**), SP (**C**) or vancomycin (**E**), or triggered with 1 μg/mL of c48/80 (**B**), 10 μM of SP (**D**) or 1 mg/mL vancomycin (**F**), for the indicated time periods. β-hexosaminidase release was determined and is presented as percentage of total. Data are the means ± SEM (n = 3–6). Statistical significance was determined by one-way ANOVA, followed by Dunnett’s post-test (* *p* < 0.05, ** *p* < 0.01, *** *p* < 0.001 and **** *p* < 0.0001).

To compare the characteristics of MRGPRX2 responses as they are expressed in RBL-MRGPRX2 versus LAD-2 cells, we selected three ligands that represent distinct categories of MRGPRX2-activating stimuli. The latter included compound 48/80 (c48/80), a synthetic polyamine, often used as the gold standard of this family [42]; substance P (SP), representing the family of MRGPRX2-activating neuropeptides [43]; and the antibiotic vancomycin, as representative of the group of FDA-approved drugs that elicit pseudo-allergic reactions by binding to MRGPRX2 [44]. Indeed, neither of these ligands triggered secretion from naïve untransfected RBL cells, consistent with their lack of expression of MrgprB3, whereas all three ligands stimulated degranulation of RBL-MRGPRX2 cells, as indicated by the release of the SG localized enzyme, β-hexosaminidase (Figure 2). These results therefore confirmed that MRGPRX2 was the exclusive mediator of secretion stimulated by these ligands in RBL-MRGPRX2 cells. Analyses of the dose–response relationships revealed that LAD-2 and RBL-MRGPRX2 cells responded to these ligands with similar affinities, i.e., EC_50_ of 0.54 versus 1.52 µg/mL of c48/80, 5.44 versus 3.07 µM of SP and 0.47 versus 0.41 mg/mL of vancomycin, respectively (Figure 2A,C,E). LAD-2 cells responded faster than RBL-MRGPRX2 cells (Figure 2B,D,F); however, this faster kinetics can be attributed to the higher level of expression of MRGPRX2 in the LAD-2 cells. Therefore, overall, our results demonstrate that RBL-MRGPRX2 and LAD-2 cells share a similar profile of responsiveness to distinct types of MRGPRX2-activating ligands.

### 3.2. ERK1/2 Signaling Is Linked with Secretion in LAD-2 and RBL-MRGPRX2 Cells

**Figure 3:**
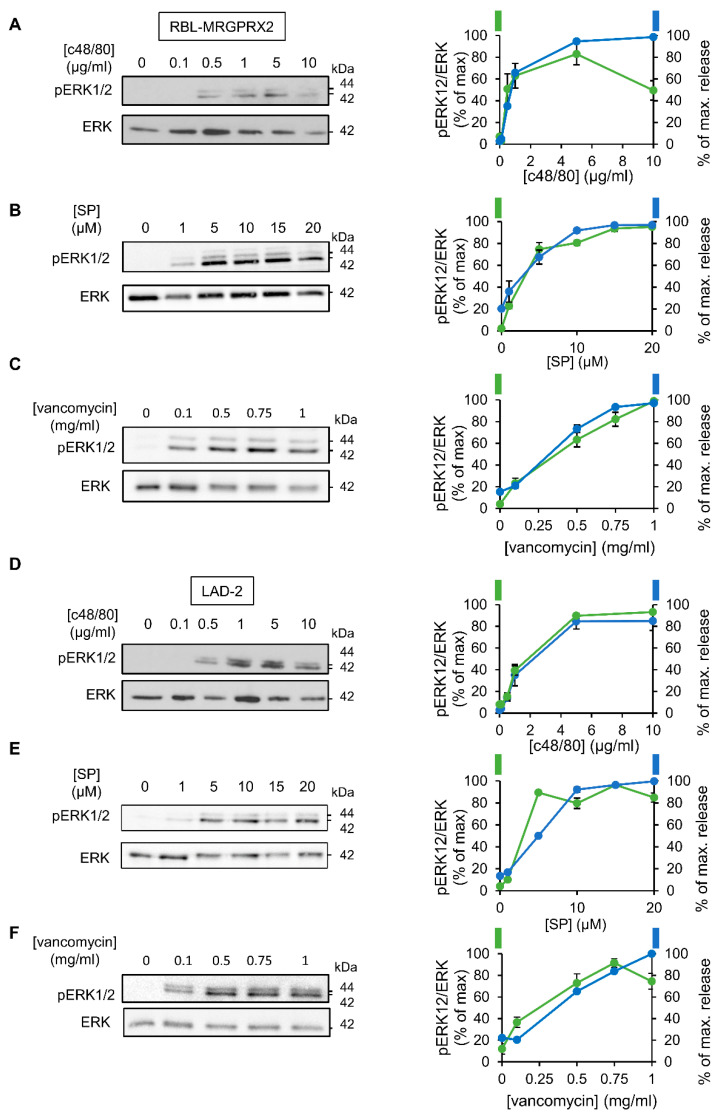
MRGPRX2-stimulated phosphorylation of the ERK1/2 MAP kinases shares similar dose–response relationships in LAD-2 and RBL-MRGPRX2 cells. RBL-MRGPRX2 (**A**–**C**) and LAD-2 (**D**–**F**) cells (1 × 10^6^ cells/mL) were incubated for 1 min, at 37 °C, with the indicated concentrations of c48/80, SP or vancomycin. Cell lysates were resolved by SDS-PAGE and immunoblotted with anti-pERK1/2 antibodies, followed by reprobing with anti-total-ERK2, as indicated. The intensities of the bands corresponding to phospho-ERK1/2 and total ERK2 were quantified by densitometry, using Image-J software, and the relative pixel densities (phosphorylated/total) were calculated and are presented as percentage of maximal response, in comparison with the percentage of the maximal secretory response at same doses. Data are means ± SEM (n = 3–4). Representative blots are shown.

**Figure 4:**
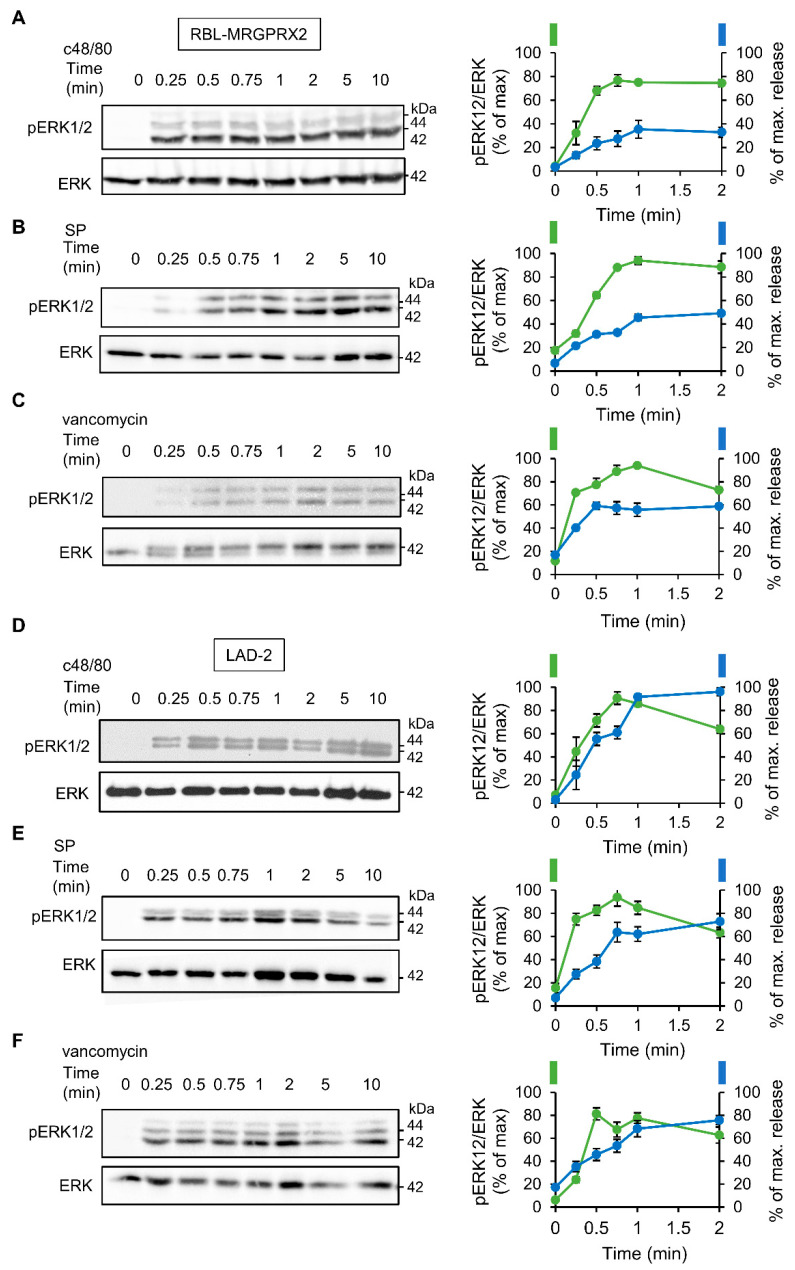
MRGPRX2-stimulated phosphorylation of the ERK1/2 MAP kinases shares similar kinetics in LAD-2 and RBL-MRGPRX2 cells. RBL-MRGPRX2 (**A**–**C**) and LAD-2 (**D**–**F**) cells (1 × 10^6^ cells/mL) were incubated with 1 µg/mL c48/80, 10 µM SP or 1 mg/mL of vancomycin, at 37 °C, for the indicated time periods. Cell lysates were resolved by SDS-PAGE and immunoblotted with anti-pERK1/2 antibodies, followed by reprobing with anti-total-ERK2 antibodies, as indicated. The intensities of the bands corresponding to phospho-ERK1/2 and total ERK2 were quantified by densitometry, using Image-J software, and the relative pixel densities (phosphorylated/total) were calculated and are presented as percentage of maximal response, in comparison with the percentage of the maximal secretory response at the same time points. Data are means ± SEM (n = 3–4). Representative blots are shown.

To investigate the relationship between signaling and secretion, we chose the MAP kinases ERK1 and 2 as reporters because of their established role in MC activation [45,46,47,48]. Comparison of the dose response relationships of ERK1/2 phosphorylation and secretion for each of the tested ligands revealed a similar dose dependency for both processes (Figure 3). Furthermore, comparing the kinetics of secretion with that of ERK1/2 phosphorylation revealed that ERK1/2 phosphorylation stimulated by either ligand, and either in LAD-2 or RBL-MRGPRX2 cells, preceded secretion (Figure 4).

### 3.3. Inhibitor Profiling of MRGPRX2-Induced Secretion from LAD-2 and RBL-MRGPRX2 Cells

**Figure 5:**
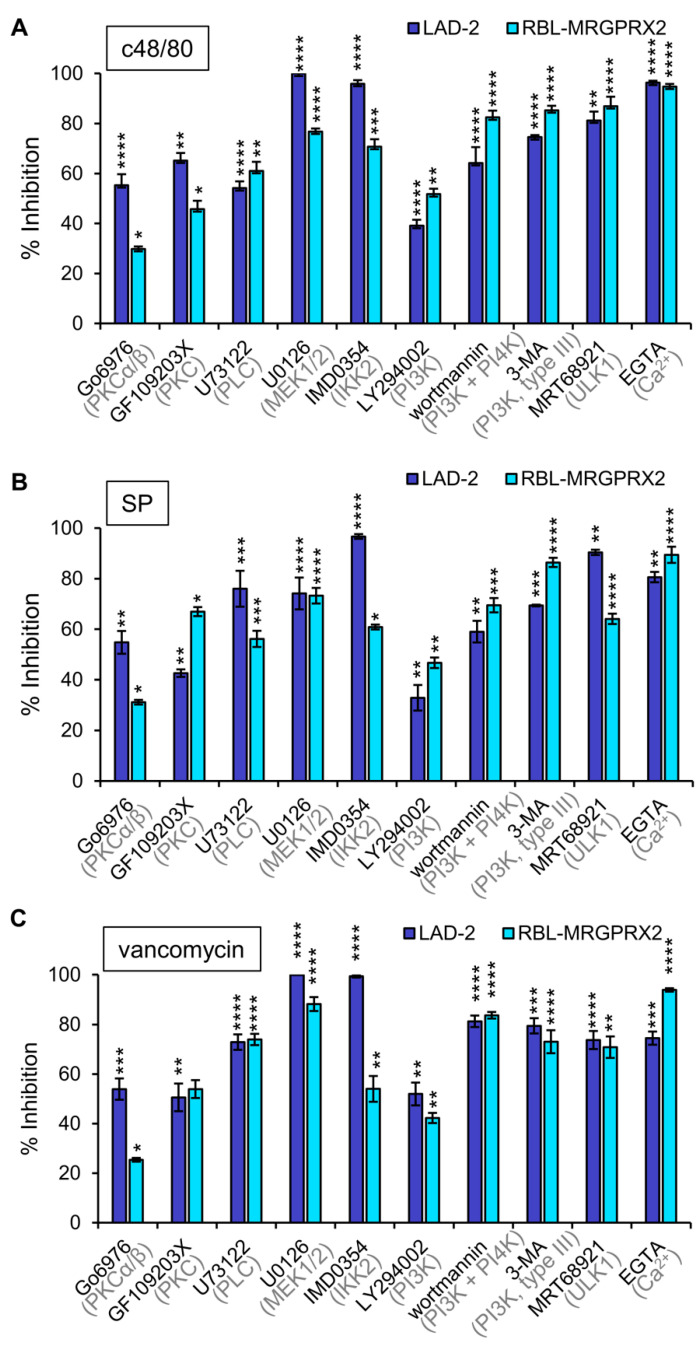
Inhibitor profiling of MRGPRX-2-induced secretion in LAD-2 and RBL-MRGPRX2 cells reveals similar patterns of drug sensitivity. RBL-MRGPRX2 and LAD-2 cells were incubated for 30 min with vehicle or 1 μM Go6976, 1 μM GF109203X, 10 μM U73122, 10 μM U0126, 0.5 μM IMD0354, 10 μM LY294002, 50 μM wortmannin, 5 mM 3-MA, 1 μM MRT68921 or 2 mM EGTA, as indicated. Cells were then stimulated by either 1 μg/mL c48/80 (**A**), 10 μM SP (**B**) or 1 mg/mL vancomycin (**C**), for a further 30 min. Release of β-hexosaminidase was determined, and percent of inhibition was calculated and compared for the two cell types, as indicated. Data are the means ± SEM of 5–10 separate experiments. Statistical significance was determined by one-way ANOVA, followed by Dunnett’s post-test (* *p* < 0.05, ** *p* < 0.01, *** *p* < 0.001 and **** *p* < 0.0001).

To investigate whether or not MRGPRX2 employs similar signaling pathways to stimulate secretion in LAD-2 and RBL-MRGPRX2 cells, we next performed a pharmacological screen, to compare the sensitivity of secretion in either cell type to inhibitors that target specific signaling pathways. We reasoned that a similar sensitivity profile would support the notion of similar signaling pathways leading to secretion by the endogenous or ectopically expressed receptor. Inhibitors chosen for this purpose were previously shown to target pathways implicated in MC responses [45,49,50,51], and included GF109203X and Go6976, inhibitors of the Ca^2+^-dependent isoforms of protein kinase C (PKC), where the latter also inhibits protein kinase D (PKD); U73122, an inhibitor of phospholipase C (PLC); the MEK inhibitor U0126, which inhibits the activation of the ERK1/2 MAP kinases; IMD0354, an inhibitor of IKKβ; LY294002, a pan inhibitor of phosphatidylinositol 3 kinases (PI3Ks); wortmannin, an inhibitor of PI3 and PI4 kinases; 3-MA, an inhibitor of type III (hVPS34) PI3K; MRT68921, an inhibitor of ULK1; and EGTA that chelates external Ca^2+^ ions. Results revealed that all inhibitors significantly reduced secretion in either LAD-2 or RBL-MRGPRX2 cells (Figure 5). Only small changes in drug sensitivity were noticed for individual ligands (Supplementary Materials Figure S1), revealing that the general pattern of drug sensitivity is conserved for distinct MRGPRX2-activating ligands. Particularly potent were the Ca^2+^ chelator EGTA and the inhibitors of ERK1/2 activation (U0126), IKKβ (IMD0354) and enzymes linked with stimulated autophagy (3-MA and MRT68921). Less potent were the inhibitors of PKC or type I PI3Ks. Therefore, these results support a unifying mechanism of action of the distinct types of MRGPRX2 ligands.

### 3.4. Inhibitor Profiling of MRGPRX2-Stimulated ERK1/2 Phosphorylation in LAD-2 and RBL-MRGPRX2 Cells

**Figure 6:**
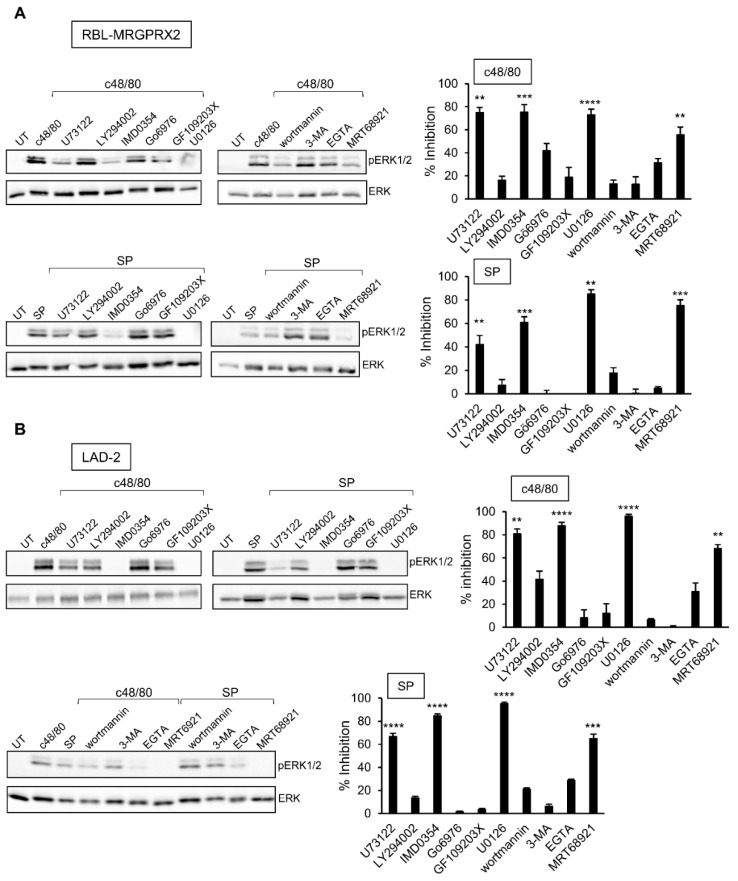
Inhibitor profiling of MRGPRX-2-stimulated ERK1/2 phosphorylation in LAD-2 and RBL-MRGPRX2 cells reveals similar patterns of drug sensitivity. RBL-MRGPRX2 (**A**) and LAD-2 (**B**) cells (1 × 10^6^ cells/mL) were incubated for 30 min, at 37 °C, with vehicle or 10 μM U73122, 10 μM LY294002, 0.5 μM IMD0354, 1 μM Go6976, 1 μM GF109203X, 10 μM U0126, 50 μM wortmannin, 5 mM 3-MA, 1 μM MRT68921 or 2 mM EGTA, as indicated. Cells were then stimulated by either 1 µg/mL c48/80 or 10 µM SP for 1 min. Cell lysates were resolved by SDS-PAGE and immunoblotted with anti-pERK1/2 antibodies, followed by reprobing with anti-total-ERK2 antibodies, as indicated. The intensities of the bands corresponding to phospho-ERK1/2 and total ERK2 were quantified by densitometry, using Image-J software, and the relative pixel densities (phosphorylated/total) were calculated. Results are presented as percent of inhibition. Data are the means ± SEM (n = 3–6). Statistical significance was determined by one-way ANOVA, followed by Dunnett’s post-test (** *p* < 0.01, *** *p* < 0.001, **** *p* < 0.0001). Representative blots are shown.

The profound inhibitory effect of the MEK inhibitor U0126 on secretion has implicated ERK1/2 signaling as critical for degranulation. Therefore, we next analyzed the relationship between this pathway and the other signaling pathways, whose targeting inhibited secretion. Expectedly, the MEK inhibitor U0126 significantly inhibited ERK1/2 phosphorylation in either LAD-2 or RBL-MRGPRX2 cells (Figure 6). Phosphorylation was also significantly inhibited by IMD0354 and MRT68921 (Figure 6), therefore positioning their cellular targets, IKKβ and ULK1, upstream of ERK1/2. Interestingly, while the PLC inhibitor of U73122 significantly inhibited ERK1/2 phosphorylation, neither the inhibition of PKC, nor chelation of Ca^2+^ by EGTA, two downstream pathways of PLC signaling, had any significant impact on ERK1/2 phosphorylation (Figure 6). Therefore, U73122 might impact ERK1/2 signaling independently of PLC, most likely via its interaction with phosphatidylinositol 4,5-bisphosphate (PIP_2_).

### 3.5. Secretion from Murine Peritoneal MCs Is Resistant to Inhibitors That Abrogate MRGPRX2-stimulated Secretion

**Figure 7:**
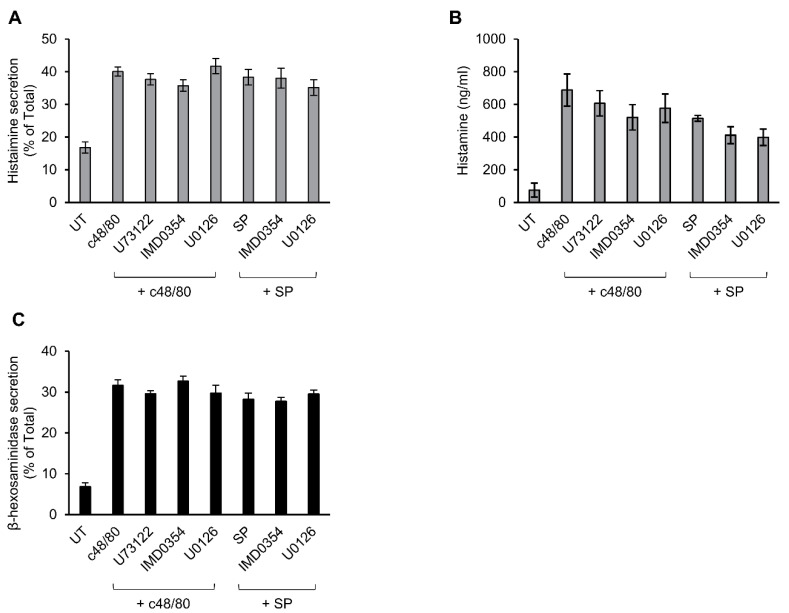
Inhibitor profiling of secretion in mouse peritoneal mast cells (MCs). Mouse peritoneal cells containing ~2 × 10^5^ MCs/mL were incubated for 30 min, with vehicle or 10 μM U73122, 10 μM U0126 or 0.5 μM IMD0354. Cells were then stimulated by either 10 µg/mL c48/80 or 100 µM SP, for a further 30 min. Secretion of histamine and β-hexosaminidase was determined. Histamine release is presented as percentage of total (**A**) and absolute amounts (**B**), which were determined by assaying histamine standards. β-hexosaminidase release is presented as percentage of total (**C**). Data are the means ± SEM (c48/80: n = 4; SP: n = 3).

Previously, we showed that c48/80-stimulated secretion of histamine from rat peritoneal MCs is resistant to inhibitors of ERK1/2 [52], implying distinct mechanisms of Mrgpr-stimulated secretion in rodent versus human MCs. To further substantiate this notion, we compared the sensitivity of secretion triggered in mouse peritoneal MCs by c48/80 or SP to inhibitors that effectively abrogated secretion in LAD-2 or RBL-MRGPRX2 cells. These experiments demonstrated that, in sharp contrast to MRGPRX2-mediated secretion, neither c48/80 nor SP-stimulated secretion of histamine in mouse MCs displayed any sensitivity to U73122, IMD0354 or U0126 (Figure 7A,B). This lack of inhibition was not a function of the type of mediator measured, because similar results were obtained when measuring release of β-hexosaminidase from the triggered cells (Figure 7C and Supplementary Figure S2). Therefore, the mechanisms utilized by rodent Mrgprs to trigger secretion are distinct from MRGPRX2-elicited responses in human MCs or when ectopically expressed in rat MCs.

## 4. Discussion

The increasing use of RBL-MRGPRX2 cells as a model for exploring the function and underlying mechanism of action of MRGPRX2 in MCs prompted us to investigate the validity of this model, in which the human receptor is ectopically expressed in rat MCs. Specifically, we compared the responses of the human cell line LAD-2, which endogenously expresses MRGPRX2, to three representative ligands of this receptor, to the responses elicited by same ligands in RBL-MRGPRX2 cells. Based on this comparative study, the following conclusions could be drawn: (1) RBL-MRGPRX2 cells target the receptor to their cell surface and respond to all three types of ligands, though their level of surface receptor is lower compared with LAD-2 cells, and, accordingly, their secretory response is slower. (2) In both LAD-2 and RBL-MRGPRX2 cells, a fraction of the receptor resides at the SGs. This is an interesting, rather unexpected, observation that may have important implications on the function of this receptor. GPCRs are known to acquire distinct patterns of signaling, depending on their location on the plasma membrane or endosomes [53]. Thus, it will be interesting to explore whether SG-localized MRGPRX2 signals differently than the cell surface receptor and whether the relative distribution of the receptor between the plasma membrane and SGs is stimulus-dependent. (3) The affinity of the receptor to its ligands is similar, whether endogenously expressed in LAD-2 cells or ectopically expressed in RBL cells. (4) MRGPRX2-stimulated secretion in LAD-2 or RBL-MRGPRX2 cells displays similar sensitivities to an array of signaling targeting drugs, implying that, despite its ectopic expression in rat MCs, MRGPRX2 preserves its authentic signaling pathways to evoke secretion. This conclusion gains further support from our results demonstrating that secretion evoked by the same ligands in mouse peritoneal MCs is resistant to the drugs that impair secretion in either LAD-2 or RBL-MRGPRX2 MCs. Therefore, taken together with our previous results, which demonstrated drug resistance of c48/80-stimulated secretion in rat peritoneal MCs to inhibitors of ERK1/2 signaling [27], our results imply distinct underlying mechanisms of Mrgpr-stimulated secretion in rodent versus human MCs. Thus, it is the receptor, and not its downstream partners, that dictates the mechanism of secretion, regardless of whether it is endogenously or ectopically expressed. Notably, these results are also compatible with our previous findings, which demonstrated that, unlike the pertussis toxin sensitivity of the Ca^2+^ responses elicited by the rodent MrgprB3 receptor, the Ca^2+^ response stimulated by MRGPRX2 is pertussis toxin insensitive. Therefore, distinct G-proteins are implicated in transmitting the Ca^2+^ signals of the rodent and human Mrgpr receptors [4]. Interestingly, unlike degranulation, stimulation of arachidonic acid metabolism by c48/80 in rat peritoneal MCs was dependent on ERK1/2 signaling, but it also required a 10-fold higher concentration of ligand [52]. This dichotomy was not detected in LAD-2 or RBL-MRGPRX2 cells, in which ERK1/2 signaling and secretion displayed a similar dose–response relationship; ERK1/2 phosphorylation preceded secretion and inhibition of ERK1/2 abrogated secretion. Therefore, while signaling by rodent MrgprB2 or MrgprB3 seems to bifurcate to elicit degranulation and metabolism of arachidonic acid, MRGPRX2 most likely utilizes a unifying mechanism for triggering both responses.

**Figure 8:**
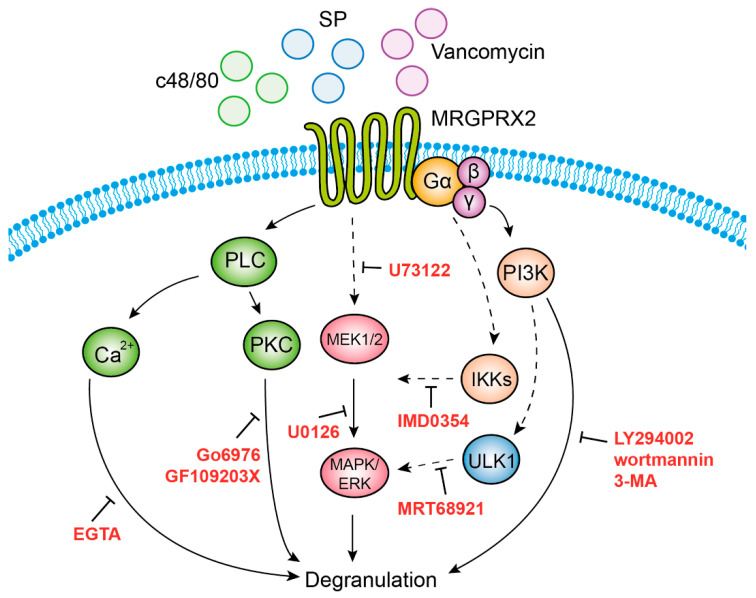
Model of MRGPRX2 stimulus–secretion coupling mechanisms. According to our model, MRGPRX2 stimulates degranulation by provoking three signaling pathways that act in concert. Activation of IKKβ leads to activation of MEK1/2 (targets of U0126), and subsequent activation of the ERK1/2 MAP kinases. Activation of PLC (target of U73122) leads to Ca^2+^ mobilization and activation of protein kinase C (PKC), whose respective targeting by EGTA, or Go6976 and GF109203X, inhibits degranulation. This signaling cluster may either be activated in parallel to ERK1/2 signaling or downstream of ERK1/2. Finally, inhibition of degranulation by 3-MA and MRT68921 implicates, in MRGPRX2-stimulated secretion, the involvement of type III phosphatidylinositol 3 kinase (PI3K) hVPS34 (target of 3-MA) and ULK1 (target of MRT68921), which are linked with the stimulation of autophagy. Our results do not exclude the involvement of type I or type II PI3Ks, which are also targets of LY294002, or PI4K, a target of wortmannin.

Our study also identified three functional clusters that couple MRGPRX2 signaling to secretion. The first cluster, which comprises ERK1/2 MAP kinase signaling, is also shared with the IgE pathway [48,51,54], implying that ERK1 and 2 regulate a central step in the exocytic process that is independent of the stimulus type. Given the short period of incubation with the MEK inhibitor U0126 and the fast kinetics of degranulation, the contribution of ERK1/2 to the secretory process is unlikely to involve transcriptional functions, such as in the case of newly synthesized mediators by activated MCs [48,51,54], nor is it likely to be due to interference with the biogenesis of the SGs that was recently shown to require ERK1/2 signaling [55]. Thus, the precise mechanism by which ERK1/2 may regulate exocytosis is presently unknown. We are inclined to position the ERK1/2-regulated step upstream or parallel to PLC signaling, the second functional cluster linked with degranulation (see model, Figure 8), as indicated by the inhibitory impact on secretion of the PLC inhibitor U73122, the inhibitors of classical PKCs (GF109203X and Go6976) and Ca^2+^ chelation. It is interesting to note that, unlike most GPCRs that normally couple to PLCβ, recent studies demonstrate that MRGPRX2 activation leads to phosphorylation of PLCγ1 [56]. However, since neither chelation of Ca^2+^ nor inhibition of PKCs affected ERK1/2 phosphorylation, the inhibitory effect of U73122 on ERK1/2 phosphorylation might be due to an indirect effect of this inhibitor, possibly by its influence on phospholipase D (PLD), which is activated by this class of stimuli [57]. Intriguingly, our results reveal a crosstalk between the ERK1/2 pathway and IKKβ, an enzyme implicated in NF-κB signaling, but also in secretion, via its mediated phosphorylation of the SNARE protein SNAP23 [58]. It is interesting to note that such an association was already demonstrated to occur in innate immune responses triggered by stimulation of Toll-like receptors, where the MAP3 kinase tumor progression locus 2 (TPL-2) functions as an MEK1/2 kinase, following its phosphorylation by IKKβ [59]. Thus, our findings extend these observations, suggesting a similar axis of signaling in mediating MRGPRX2-stimulated responses. Finally, the third cluster encompasses autophagy-related signaling, as evidenced by the inhibition of secretion by drugs that target enzymes involved in the induction or execution of the autophagic process. Included are the pan inhibitors of PI3Ks LY29004 and wortmannin, 3-MA, which targets the type III PI3K, hVPS34 and MRT68921, an inhibitor of the autophagy-inducer ULK1. These findings join earlier observations that already alluded to a crucial role for autophagy in MC degranulation [60,61]. Furthermore, consistent with previous reports that associated the degree of ERK phosphorylation with the abundance of autophagic structures [62], our results depict some crosstalk between these pathways, whereby inhibition of ULK1 inhibited ERK1/2 phosphorylation. 

In conclusion, we show that RBL-MRGPRX2 cells recapitulate the underlying mechanisms of MRGPRX2-stimulated degranulation in human MCs and provide new insights into the underlying mechanism of MRGPRX2-stimulated secretion. Finally, our results demonstrate the distinct species-dependent features of secretion evoked by Mrgprs, therefore calling for caution when applying results of studies in rodent animal models to humans. 

## Figures and Tables

**Table 1:** List of drugs used in this study.

Inhibitor	Target	Company	Cat. No.
Go6976	PKCα/β_1,_ PKD	A.G. Scientific (San Diego, CA, USA)	G-1017
GF109203X	PKC	A.G. Scientific	G-1063
U73122	PLC (+PLD)	TOCRIS (Minneapolis, MN, USA)	1268
LY294002	PI3K	TOCRIS	1130
wortmannin	PI3K + PI4K	A.G. Scientific	W-1022
U0126	MEK1, MEK2	A.G. Scientific	U-1026
MRT68921	ULK1/2	TOCRIS	5780
IMD0354	IKKβ	Cayman Chemical (Ann Arbor, MI, USA)	17290
3-MA	PI3K type III	Sigma-Aldrich	M9281
EGTA	Ca^2+^ chelator	Sigma-Aldrich	E4378

PKD, protein kinase D; PLC, phospholipase C.

**Table 2:** Primers used in this study.

hMRGPRX2 (PrimerBank ID40255006c1) [32,33,34]	fw 5′-CTGGTAGGAAACGGGTTTGTG-3′ rv 5′-GCTGAGGACGTAGACAGAGAAG-3′
rMRGPRB3 [35]	fw 5′-CCCCTGGAATGTTCTTTTGTGTAG-3′ rv 5′-ACAGTGAAAAATGCAGGAACTTGG-3′
hGAPDH (cat #: HP205798, sequence from Origene, ordered from Hy Laboratories Ltd., Rehovot, Israel)	fw 5′-GTCTCCTCTGACTTCAACAGCG-3′ rv 5′-ACCACCCTGTTGCTGTAGCCAA-3′
rGAPDH [36]	fw 5′-TGGAGTCTACTGGCGTCT-3′ rv 5′-TGTCATATTTVTCGTGGT-3′

## Data Availability

No datasets were generated or analyzed during this study.

## Supplementary Materials

The following are available online at https://www.mdpi.com/2073-4409/10/2/376/s1. Figure S1: Inhibitor profiling of MRGPRX-2-induced secretion in LAD-2 and RBL-MRGPRX2 cells. Figure S2: Comparison of inhibitor sensitivity of β-hexosaminidase secretion from mouse peritoneal MCs (PMCs) and RBL-MRGPRX2 and LAD-2 cells.
