# Supplementary figures and images for: Authentic and Ectopically Expressed MRGPRX2 Elicit Similar Mechanisms to Stimulate Degranulation of Mast Cells
Source: Cells. 2021 Feb 12;10(2):376. doi: 10.3390/cells10020376 (PMC7918488; doi:10.3390/cells10020376)

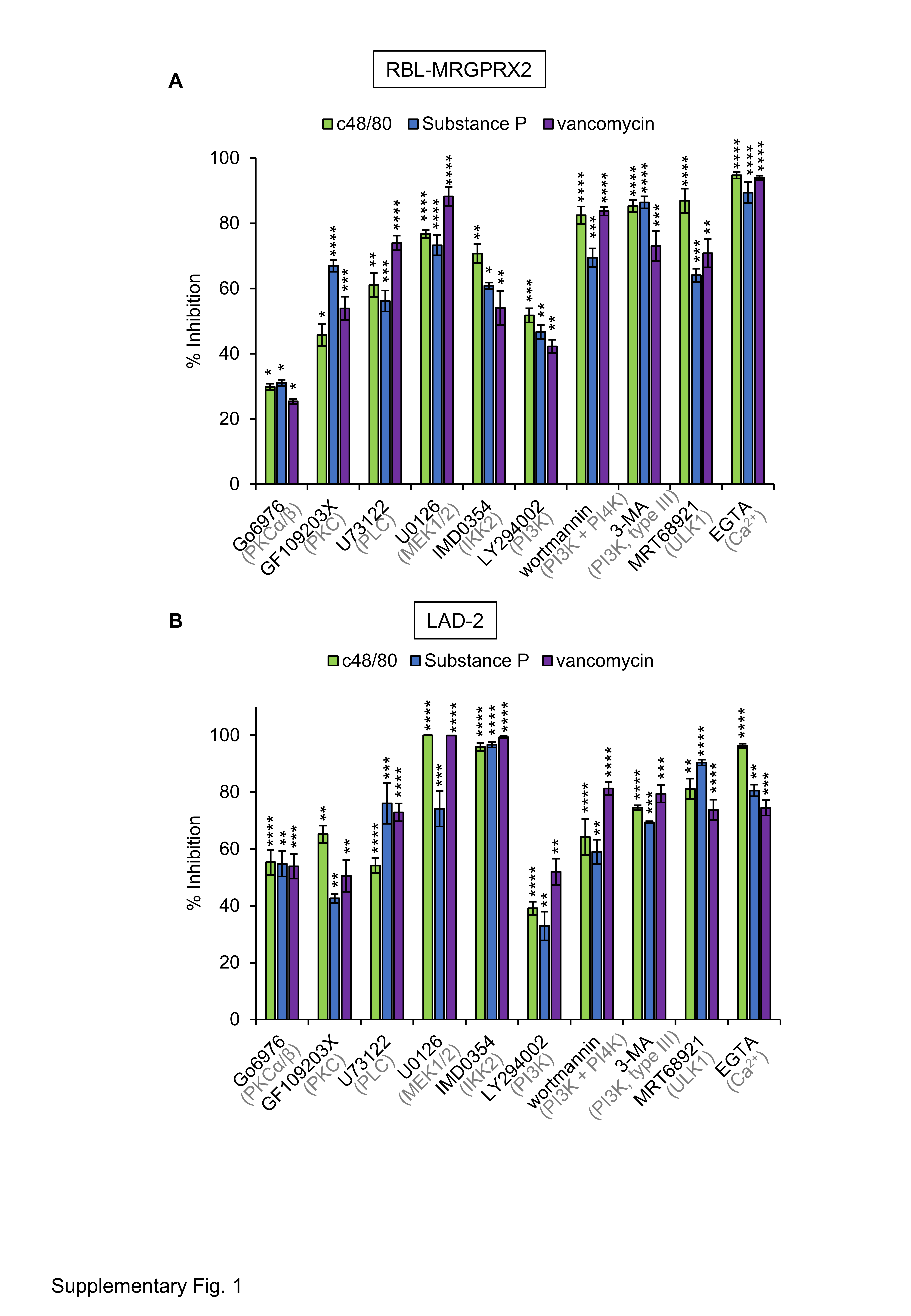

Supplement: Supplementary file 1 [file cells-10-00376-s001.zip › Supplementary Figure 1.TIF]

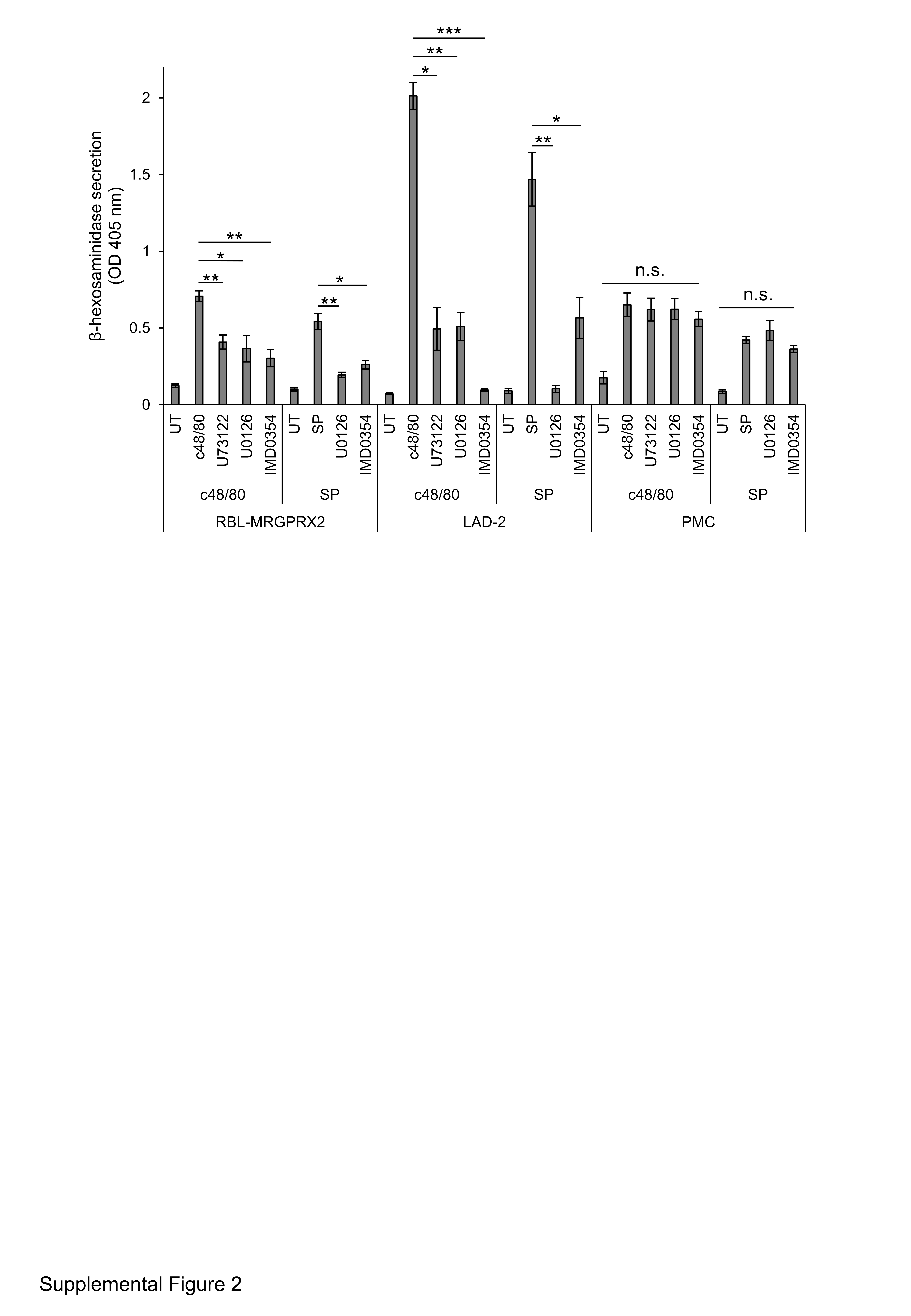

Supplement: Supplementary file 1 [file cells-10-00376-s001.zip › Supplementary Figure 2.TIF]
